# Identification of TCE and PCE sorption and biodegradation parameters in a sandy aquifer for fate and transport modelling: batch and column studies

**DOI:** 10.1007/s11356-015-4156-9

**Published:** 2015-02-04

**Authors:** E. Kret, A. Kiecak, G. Malina, I. Nijenhuis, A. Postawa

**Affiliations:** 1 Department of Hydrogeology and Engineering Geology, AGH University of Science and Technology, Krakow, Poland; 2 Department of Isotope Biogeochemistry, Helmholtz Centre for Environmental Research–UFZ, Leipzig, Germany

**Keywords:** Sorption, Biodegradation, Trichloroethene, Tetrachloroethene, Numerical modelling, Batch tests, Column tests, Isotopic measurements

## Abstract

The main aim of this study was to determine the sorption and biodegradation parameters of trichloroethene (TCE) and tetrachloroethene (PCE) as input data required for their fate and transport modelling in a Quaternary sandy aquifer. Sorption was determined based on batch and column experiments, while biodegradation was investigated using the compound-specific isotope analysis (CSIA). The aquifer materials medium (soil 1) to fine (soil 2) sands and groundwater samples came from the representative profile of the contaminated site (south-east Poland). The sorption isotherms were approximately linear (TCE, soil 1, *K*_d_ = 0.0016; PCE, soil 1, *K*_d_ = 0.0051; PCE, soil 2, *K*_d_ = 0.0069) except for one case in which the best fitting was for the Langmuir isotherm (TCE, soil 2, *K*_f_ = 0.6493 and *S*_max_ = 0.0145). The results indicate low retardation coefficients (*R*) of TCE and PCE; however, somewhat lower values were obtained in batch compared to column experiments. In the column experiments with the presence of both contaminants, TCE influenced sorption of PCE, so that the *R* values for both compounds were almost two times higher. Non-significant differences in isotope compositions of TCE and PCE measured in the observation points (δ^13^C values within the range of −23.6 ÷ −24.3 ‰ and −26.3 ÷−27.7 ‰, respectively) indicate that biodegradation apparently is not an important process contributing to the natural attenuation of these contaminants in the studied sandy aquifer.

## Introduction

Dissolved chlorinated solvents, such as trichloroethene (TCE) and tetrachloroethene (PCE), are among the most frequently detected organic contaminants in groundwater worldwide and thus pose a significant threat to drinking water supply systems (Stroo and Ward 2010; Wiedemeier et al. 1999). After the Water Framework Directive (2000/60/EC) had been implemented into the Polish law, consequently, new drinking water standards were applied; hence, a number of sites in Poland groundwater contamination of TCE and PCE were detected (Kret et al. 2013). Consequently, there is an increasing need to investigate these sites with a special emphasis on description and prediction of the contaminants’ transport and fate in groundwater systems from the source to the defined receptor(s). This can be facilitated by using numerical modelling, representing the physical phenomena occurring in groundwater systems (Bear and Cheng 2010; Van der Heijde et al. 1980). Numerical modelling is a valuable tool for simulating contaminants’ migration along the transport pathway, risk assessment, as well as planning and designing effective groundwater remediation systems (Kitanidis and McCarty 2012). However, a successful model development relies largely on appropriate estimates for the aquifer parameters (Rifai et al. 2010). Such parameters can be obtained with a series of experimental methods, estimated using prediction methods or found in the literature (Dello Site 2001). Parameters’ estimation is particularly important and may be difficult in the case of TCE and PCE, as they belong to DNAPLs (dense non-aqueous phase liquids) and may sink at the bottom of an aquifer as a separate phase and then spread out in the direction of groundwater flow along the preferential pathways (Kueper et al. 2003).

The aim of this paper is to estimate sorption (using batch and column tests) and biodegradation (by means of the compound-specific isotope analysis—CSIA) as the input parameters for a numerical fate and transport model of TCE and PCE in the sandy aquifer in south-east Poland, where their concentrations in groundwater exceed drinking water standards more than 1000 times (Kret et al. 2011). Such model is currently being developed and applied to assess the risk associated with TCE and PCE contamination, as well as to select and design the effective groundwater remediation strategy.

### Transport parameters

To fully represent TCE and PCE migration in a numerical model, it is required to know physicochemical properties of the contaminants, as well as hydrogeological and geochemical conditions of the site, such as hydraulic conductivity (*k*), hydraulic gradient (*H*), dry bulk density (*ρ*_s_), porosity/effective porosity (*θ*_t_/*θ*), longitudinal and transverse dispersivity (*α*_L_, *α*_v_), distribution coefficient (*K*), biodegradation rate (usually incorporated in the model as a first order decay), initial concentrations (*C*_s_) and source size (Strickland and Korleski 2007). Along the transport pathway, a variety of physical, chemical and/or biological processes may influence contaminants’ migration (Dridi et al. 2009; Wiedemeier et al. 1999), from which the most important are dispersion, dilution, volatilization, sorption and biodegradation. The last two mostly influence the TCE and PCE migration velocity in an aquifer (Dridi et al. 2009). All of these processes are incorporated to the model by single or a set of governing equations. Three dimensional transports of contaminants in groundwater, with constant porosity, can be described by the partial differential equation (Zheng and Wang 1999):

1 $$ \frac{\partial C}{\partial t}=\frac{\partial }{\partial {x}_i}\left[{D}_{ij}\frac{\partial C}{\partial {x}_j}\right]-\frac{\partial }{\partial {x}_i}\left({v}_iC\right)+\frac{q_s}{\theta }{C}_s+{\displaystyle \sum {R}_n} $$

where *C*—the concentration of contaminant dissolved in groundwater (ML^−3^); *t*—time (t); *x*_*i*_—the distance along the respective Cartesian coordinate axis (L); *D*_*ij*_—the hydrodynamic dispersion coefficient tensor (L^2^T^−1^); *v*_*i*_—the seepage or linear pore water velocity (LT^−1^); *q*_*s*_—the volumetric flow rate per unit volume of aquifer representing fluid sources (positive) and sinks (negative) (T^−1^); *C*_*s*_—the concentration of the source or sink flux for contaminant (ML—^3^); θ—porosity of the porous medium (−); *R*_*n*_—the chemical reaction term, in this example representing sorption (retardation factor *R*) and first-order rate reaction (*λ*) (ML^−3^ T^−1^)

### Sorption

**Fig. 1:**
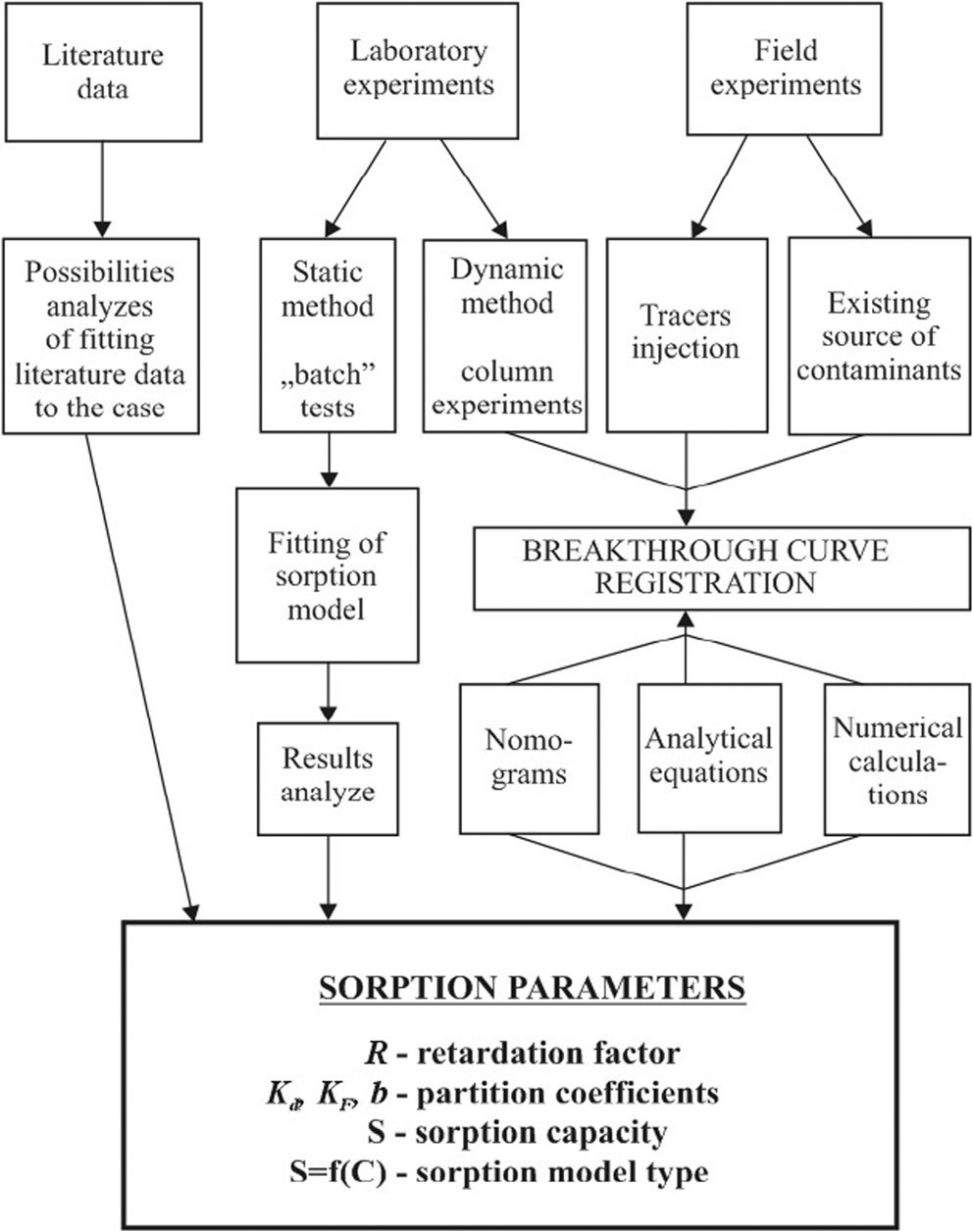
Methods of determining the sorption parameters (Dowgiałło 2002, modified)

The term sorption refers to physical and/or chemical attachment of a compound to a solid surface, i.e. porous medium solid (Abulaban et al. 1998). Sorption parameters may be estimated based on (Fig. 1) the literature, laboratory experiments (including batch and column tests) or field investigations. In the case of organic contaminants, the most common sorption isotherms are (Table 1) Henry’s (linear), Freundlich’s (logarithmic) and Langmuir’s (including the maximum sorption capacity) (Limousin et al. 2007). To characterize TCE and PCE sorption for higher concentrations, the linear model can be used, while for lower concentrations, the Freundlich model usually fits better (Akyol et al. 2011). An appropriate sorption model may be chosen by fitting a theoretical curve to experimental data, while the accuracy of fitting may be evaluated by a determination coefficient (*r* ^2^) (Karickhoff et al. 1979). Sorption models, however, do not include the effects of speciation, pH and redox potential, thus the results of batch experiments should be regarded as approximates. Column experiments are more widely used to estimate sorption parameters by fitting breakthrough curve (BTC) (Tang et al. 2009). Flow through techniques have the advantage of approximating real conditions more closely, i.e. by allowing the solids to be rested relative to the mobile solutes and by maintaining appropriate solid/solution ratios (Benker et al. 1998). Sorption of TCE and PCE depends mainly on the organic carbon content and the aquifer material’s grain-size distribution (the content of clay and silt fractions). It is also controlled by the geochemical conditions (Cwiertny and Scherer 2010; Kueper et al. 2003).

**Table 1:** Selected sorption models (Hinz 2001; Limousin et al. 2007)

Sorption model	Formula	Isotherm’s type	Retardation factor (*R*)
Linear (Henry’s)	*S* = *K*_*d*_ ⋅ *C*		$R=1+\frac{\rho_d}{\theta }{K}_d$
Freundlich	*S* = *K* ⋅ *C* ^*η*^		$R=1+\frac{\rho_d\cdot \eta \cdot {C}^{\eta -1}}{\theta}\cdot K$
Langmuir	$\frac{S}{S_{\max }}=\frac{K\cdot C}{1+K\cdot C}$		$R=1+\frac{\rho_d\cdot {S}_{\max }}{\theta \cdot {\left(1+K\cdot C\right)}^2}\cdot K$

*C* aqueous pollutant concentration [mg/L], *S* mass of pollutant adsorbed to mass of adsorbent [mg/kg], *S*_max_ maximum sorption capacity [mg/kg], *K, K*_*d*_ partition coefficients, *η* degree of isotherm’s nonlinearity characterizing adsorption energy, *ρ*_*d*_ bulk density, *θ* effective porosity

Sorption parameters have already been tested in several studies using different methods and diverse soil types. The list of results presented in the literature with references is shown in Table 2. Authors usually postulate that for sandy materials, the retardation factor ranges from 1.04 to 2.95 (TCE) and from 1.2 to 3.6 (PCE) depending on TOC content, soil type, and the type of experiment.

**Table 2:** Comparison of sorption parameters of TCE and PCE on sandy materials obtained in diverse laboratory studies

Soil type	TOC [%]	Compound	Sorption parameters^a^		Isotherm	*R*	Method	Reference
Medium sand	0.52	TCE	0.0016		Henry’s	1.01	Batch	this study
		PCE	0.0051			1.31		
		TCE	–		–	1.0–1.11	Column	
		PCE				1.11–1.39		
Fine sand	0.32	TCE	0.0145	0.6493	Langmuir	1.34	Batch	
		PCE	0.0069		Henry’s	1.41		
		TCE	–		–	1.14–1.43	Column	
		PCE				1.28–1.54		
Sand	0.03	PCE	0.23	0.90	Freundlich	–	Batch	Brusseau et al. 2012
Sand	0.38		1.8	0.85				
			1.3^b^	0.94				
Caliche soil	0.97	TCE	18.95		–	1.75–2.95	Column	Akyol et al. 2011
Silty sand	–	TCE	0.375–0.639		Henry’s	–	Batch	Jo et al. 2010
Gravel with sand and silt^c^	0.262	PCE	3.84		Henry’s	–	Batch	Ruffino and Zanetti 2009
		TCE	2.14					
Sand	0.22	PCE	0.72 ± 0.03		Henry’s	–	Batch	Ma et al. 2007
	0.54		2.20 ± 0.06					
Sand	<0.08	TCE	–		–	1.04 ± 0.05	Column	Rüttinger et al. 2006
Gravel	0.0005–0.001	TCE	–		–	1.2–2.2	–	Schuler et al. 2006
		PCE				1.4–3.4		
Fine to silty sands	0.15	TCE	0.16		Henry’s	–	Batch	Hellerich and Nikolaidis 2005
		PCE	0.49				Batch	
Fine sand	0.013	PCE	1.144		Henry’s	–	Batch	Zhao et al. 2005
Medium sand	0.040		0.451					
	0.042		0.634					
Coarse sand	0.126		2.162					
Fine to medium sand	0.0211	TCE	0.10	0.90	Freundlich	1.1–1.4	Batch	Rivett and Allen-King 2003
			0.41	0.92	Freundlich	1.9–3.6	Batch	
Gravel	<0.001	TCE	–		–	1.3–1.9	Column	Salaices Avila et al. 2002
Sand	0.13 ± 0.05	TCE	0.052 ± 0.025		Henry’s	1.2–1.5	Batch	Benker et al. 1998
			<0.008		–	<1.05	Column	
Sand	0.02	PCE	–		–	2.1–2.2	Column	Brusseau 1992
Sand	0.03	PCE	–		–	2.2–2.3		
Sand	0.007	PCE	–		–	2.1	Column	Brusseau et al. 1991; Bourg et al. 1993
		TCE				1.4–1.6		
Sandy soil solids	0.02–0.22	PCE	–		–	2.5	Column	Wilson et al. 1981; Bourg et al. 1993
		TCE				1.5–1.6		
Sand aquifer	0.007	PCE	–		–	2.2	Column	Larsen et al. 1989; Bourg et al. 1993
		TCE	–		–	1.5		
Sand aquifer	0.025	PCE	–		–	1.2		
		TCE	–		–	1.1		
Sand aquifer	0.015	PCE	–		–	1.2		
		TCE	–		–	1.1		
Medium sand	0.02	PCE	–		–	3.6 ± 0.3	Batch	Curtis et al. 1986; Bourg et al. 1993

*TOC* total organic carbon

^a^Dual-mode model: for Henry isotherm, *K*_*d*_; for Freundlich isotherm, *K*_*f*_ and *η*; for Langmuir isotherm, *S*_max_ [mg/kg] and *K*_l_

^b^In the presence of co-solutes, < 2 mm was used

^c^In the experiment soil of the grain size < 2 mm was used

### Biodegradation

Biodegradation is a process that leads to reduction of TCE and PCE loads in groundwater. It involves the breakdown of organic compounds, either through biotransformation into less complex metabolites, or through mineralization into inorganic minerals (Singh and Ward 2004; Wiedemeier et al. 1998). TCE and PCE may be biodegraded firstly by reductive dechlorination in anaerobic conditions or secondly by co-metabolism in aerobic conditions. Whether the biodegradation occurs at the specific site depends on numerous environmental conditions (pH, temperature, Eh, etc.). In the literature (e.g. Van Breukelen et al. 2005; Suarez and Rifai 1999), the wide range of TCE and PCE biodegradation rates is presented. For instance, the first-order rate constant amounts to 1.1 ÷ 3.7 and 0.9 ÷ 1.5 1/year for PCE and TCE, respectively. It is recommended to use multiple methods providing more than one line of evidence for the biodegradation assessment (Nijenhuis et al. 2007).

To assess the rates of TCE and PCE biodegradation, several methods can be used, e.g. (Bombach et al. 2010) geochemical analyses, microbial and molecular methods, tracer tests, metabolite analysis, compound-specific isotope analysis (CSIA) and in situ microcosms. Recently, stable isotope fractionation approaches have been developed and implemented at many sites (e.g. Imfeld et al. 2008; Nijenhuis et al. 2007, 2013). The CSIA takes advantage that during biodegradation, molecules with lighter isotopes (e.g. C^12^) react preferentially over the heavier isotope (C^13^) fractions, which results in an enrichment of the heavier stable isotope in the residuum (Cichocka et al. 2007). The isotope composition and residual concentration are analysed, and enrichment in the heavy isotope indicates biodegradation (Imfeld et al. 2008). The carbon isotope composition is reported in δ notation (‰) relative to the Vienna Pee Dee Belemnite standard (V-PDB, IAEA-Vienna) (Hunkeler et al. 2008):

2 $$ {\updelta}^{13}\mathrm{C}\left[{\mbox{\fontencoding{U}\fontfamily{wasy}\selectfont\char104}} \right]=\left(\frac{{\left({}^{13}\mathrm{C}/{}^{12}\mathrm{C}\right)}_{\mathrm{sample}}}{{\left({}^{13}\mathrm{C}/{}^{12}\mathrm{C}\right)}_{\mathrm{standard}}}\right)\times 1000 $$

Based on the change in isotope ratio, the biodegradation rate constant may be calculated, what is the input parameter in contaminants’ transport modelling. The isotope fractionation may be incorporated into reactive transport models and used for verification or calibration (Van Breukelen et al. 2005; Hunkeler and Aravena 2010).

## Materials and methods

### Materials

Two types of the aquifer materials for the sorption parameters estimation were collected from a representative profile at the studied site, namely medium (soil 1) and fine (soil 2) sands. Physicochemical properties of the aquifer materials were analysed and the results are listed in Table 3.

**Table 3:** Physicochemical properties of the aquifer material used in the experiments

Sample	Type	Depth [m bgl]	Total organic carbon (TOC) [%]	Βulk density *ρ*_d_ [g/mL]	Effective porosity *n*_e_ [−]
Soil 1	Medium sand	15.0 – 25.5	0.52	1.8	0.3
Soil 2	Fine sand	11.5 – 15.0	0.32	1.8	0.3

**Fig. 2:**
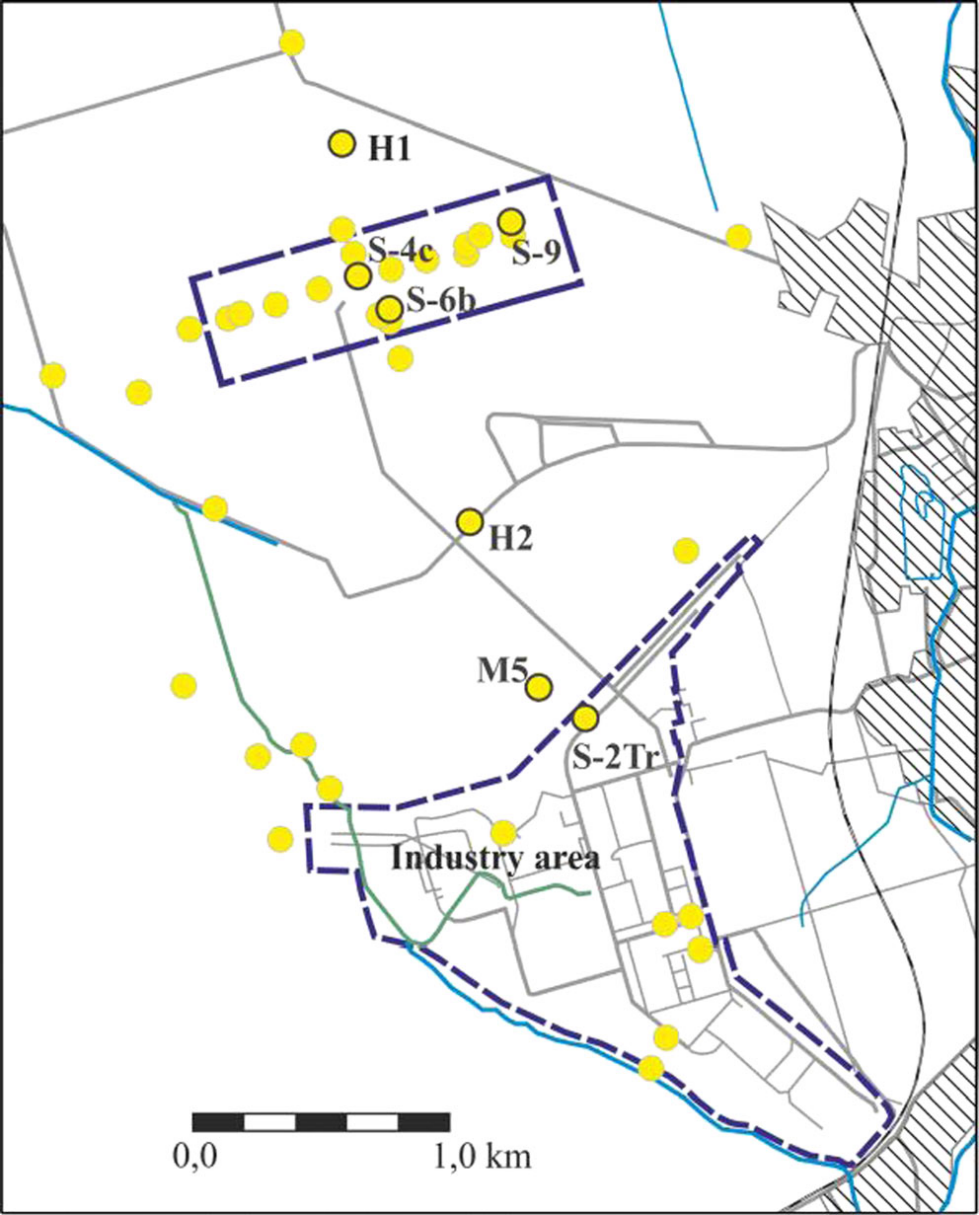
Location of monitoring wells

For the biodegradation study, seven sampling points (piezometers or wells) were chosen, located at the site along the contamination plume pathway, starting from the potential source of TCE and PCE (the former metalwork). The location of the wells is presented in Fig. 2. Groundwater samples were taken using a peristaltic pump (Ejkelkamp). To ensure representative sampling, field parameters (pH, redox potential, conductivity, temperature and dissolved oxygen content) were monitored in situ. The samples were taken after obtaining stabilization of the parameters mentioned above. Two replicate samples were collected in autoclaved 250-mL glass vials containing ca. 90 g of solid NaCl (to inhibit further microbial activity) with ca. 50-mL headspace. The samples were sealed with Teflon-coated septa.

### Batch tests

The experiment was conducted for both aquifer materials and proceeded to two stages. During the first stage, the time needed to reach equilibrium for both contaminants between sorbed and dissolved phases was estimated. The aim of the second stage was to estimate partition coefficients (*K*) based on identified sorption isotherms and to calculate retardation factors (*R*) using an appropriate formula (see Table 1).

Samples of 5.0 g (±0.1 g) of dry, uncontaminated aquifer material were placed into 50-mL borosilicate glass vials. Then, the model solutions of distilled water spiked with TCE and/or PCE were added to obtain the contaminated water (*L*) to solid phase (*S*) ratio of L/S = 5:1 (*v*/*v*). To exclude possible biodegradation (biotransformation) of TCE and PCE, 0.15 g/L of sodium azide (NaN_3_) was added. Bottles were closed immediately after filling, so that no headspace was left. Vials were shaken for 72 h with 450 rph and placed afterwards in a dark place to reach the equilibrium. The experiment was conducted at room temperature (ca. 22 °C).

In the first stage, TCE and PCE concentrations (Table 4) were selected based on their maximum values observed in groundwater at the contaminated site in 2010. TCE and PCE concentrations (depending on the sample) were measured at specified time intervals to determine time of reaching the equilibrium. The equilibrium was assumed to be reached when a difference between two successive TCE and PCE concentration measurements in the solutions does not exceed ±15 % (a measurement’s error for the detection method used, i.e. gas chromatography).

**Table 4:** Contaminants’ concentrations used in the batch tests

	Concentration [mg/L]					
	First stage	Second stage				
TCE						
Soil 1	5.04	1.17	1.74	2.30	2.86	5.04
Soil 2	4.07	–	1.04	1.80	3.10	4.07
PCE						
Soil 1	1.84	0.77	0.89	0.91	1.12	1.84
Soil 2	–	0.21	0.43	0.90	1.45	2.16

In the second stage, sorption isotherms and partition coefficients were estimated and, consequently, retardation factors *R* were calculated. Five solutions with different concentrations of TCE and PCE were prepared for both soils (Table 4), analogously as at the first stage. Samples were measured for TCE and PCE concentrations in the solutions after reaching the equilibrium (as determined in the first stage). TCE and PCE concentrations were measured using gas chromatography method (GC) at WESSLING Polska sp. z o.o. licenced laboratory in Krakow, Poland, according to PN-EN ISO 10301:202 and EN 1484 standards.

The sorbed mass of TCE/PCE related to the mass of a sorbent (aquifer material) was calculated from the equation based on mass balance:

3 $$ S=\left({C}_0-{C}_{\mathrm{eq}}\right)\cdot V/m $$

where *S*—the concentration of a contaminant sorbed by an aquifer material (mg/kg), *C*_0_—the initial concentration of a contaminant in solution (mg/L), C_eq_—the concentration of a contaminant in solution after reaching the equilibrium (mg/L), *V*—volume of solution used in the experiment (L), *m*—mass of sorbent (aquifer material) (g).

From the estimated partition coefficients (*K*) retardation factors (*R*) were calculated using the equations from Table 1 selected based on determination coefficients (*r* ^2^) of the fitted sorption models.

### Column experiment

A soil sample was placed in a special cylinder made from galvanized steel (length of 10.8 cm and 6.35 cm in diameter) and carried out under saturated conditions. The columns were equipped with a vent and filled in with the wet aquifer material progressively from the bottom of the column to avoid trapping air bubbles. Above and below the material a paper filter and steel wool were placed to avoid clogging of the inlet and outlet ports.

**Fig. 3:**
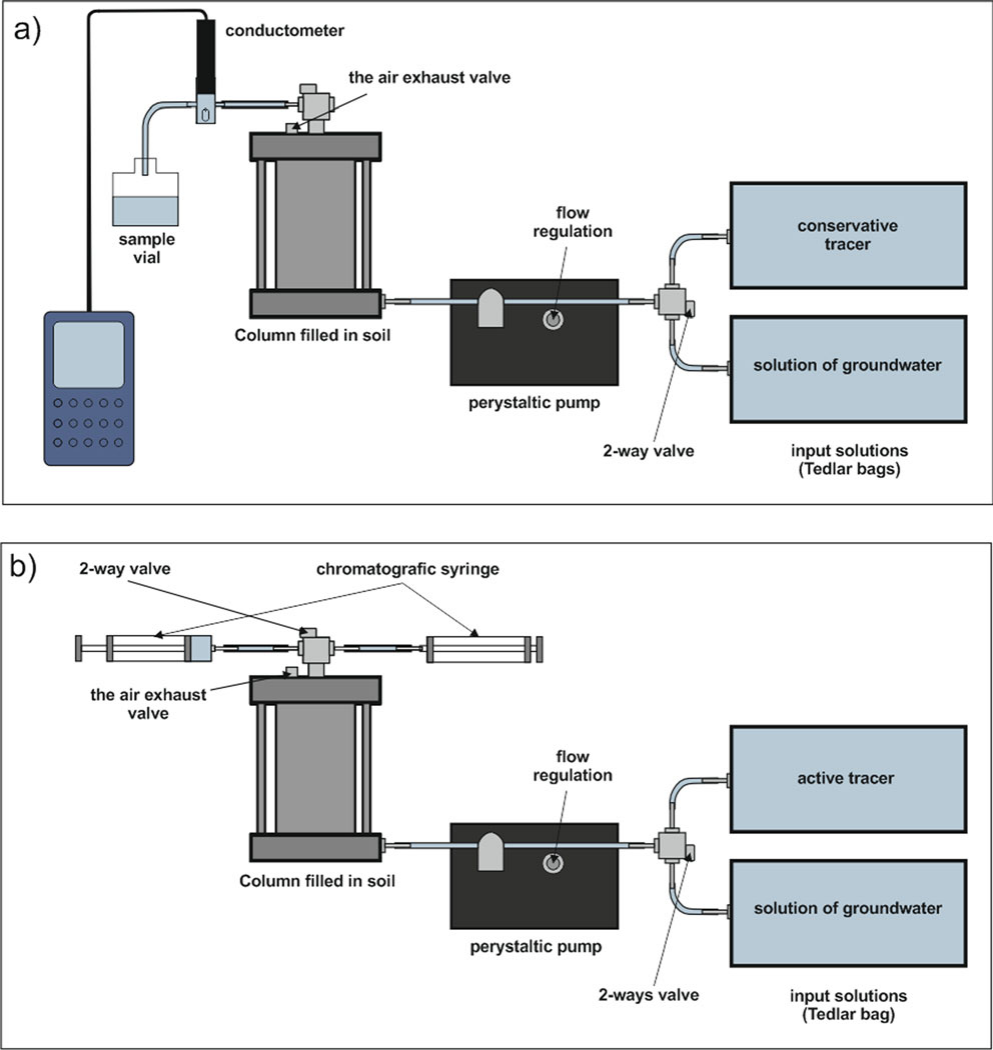
The experimental setup: **a** stage 1, **b** stage 2

Water/solution was provided to the column by a peristaltic pump with a velocity of 0.102–0.114 cm/min, similar to the actual groundwater flow rate at the site. The conservative/reactive tracers were introduced to the system by short, 5 min, injections. The system was equipped with a valve allowing for fast changes between tracer and water injections. The installation was set up to minimize migration pathways between its parts. The experiment was conducted at room temperature (ca. 22 °C). The experimental setup is shown in Fig. 3.

Groundwater from an uncontaminated well at the studied area was used with 15 g/L of sodium azide (NaN_3_) added to avoid TCE/PCE biodegradation (biotransformation). Chloride was used as a conservative tracer, while TCE and PCE served as reactive tracers. The chloride input concentration was of 500 mg/L, while TCE or/and PCE concentrations were selected based on the actual values measured in groundwater at the site (about 4 and 3 mg/L, respectively).

The experiment was conducted for both materials (soil 1 and 2) and proceeded in two stages. Throughout the first stage, the conservative tracer was injected. Before that, an equilibrium was reached in the column between the solution and sorption complex through stabilization of conductivity. Subsequently, the conservative tracer was injected and again, the valve was changed. Concentrations of the conservative tracer were measured in the outlet solution at specific times. Moreover, for the duration of the first stage an online conductivity measurement was carried out. The time of total water exchange in the column was also determined as approximately 1 h. The second stage was conducted analogously to the first one, except that the reactive tracers (TCE and PCE) were injected. The outlet solution was collected every 8 min (minimum time to reach the volume of 20 mL required for laboratory analyses). The second stage ran for 2 h, i.e. two times longer than the first stage.

The interpretation was based on breakthrough curves (BTC) showing changes of contaminant (reactive tracer) concentrations in the output solution in the function of time. Based on observed differences in conservative/reactive tracer concentrations in output solutions, it was possible to estimate the *R* coefficient. The CXTFIT/Excel was used to determine BTCs using equilibrium convection-dispersion equation (CDE) (Tang et al. 2010). In column experiment, a size of the column can influence on sorption parameters’ uncertainty, but it has no significant influence on estimation of *R* coefficient (Marciniak et al. 2006). Despite the small column diameter used in the experiment, estimation of *R* factor based on fitted BTC was done with sufficient accuracy.

TCE and PCE concentrations were measured using gas chromatography method (GC) as described in “Batch tests”. Concentration of chlorides were measured by the titration method.

### Biodegradation studies

To assess TCE and PCE biodegradation rates, CSIA was applied. Concentrations of TCE and PCE in groundwater samples were analysed with gas chromatography with flame ionization detection (GC-FID) (Varian Chromack CP-3800 equipped with a 30 m × 0.53 mm GS-Q column, J&W Scientific). The carrier gas was helium; the FID was operated at 250 °C. The injection was automated using an HP 7694 headspace auto sampler (Hewlett Packard) adding 0.5-mL headspace to 10-mL vials flushed with helium, closed with a Teflon-coated butyl rubber septum and crimped. Gas chromatography combustion isotope ratio mass spectrometer (GC-C-IRMS) was applied in order to determine the stable carbon isotope composition of the chlorinated ethenes as described previously (e.g. Imfeld et al. 2008; Kästner et al. 2010). The GC-C-IRMS system consisted of a gas chromatograph (model 6890, Agilent Technology) coupled via Conflow III interface (ThermoFinnigan) to a MAT 252 mass spectrometer (ThermoFinnigan). Samples were measured in at least three replicates. The carbon isotope ratio measurements were conducted via headspace analysis. Aliquots (500 to 1000 μL) of headspace samples were injected into a gas chromatograph in splitless or split mode (split 1:1) using split/splitless injector at 250 °C. Column used for the separation was PoraBOND (50 m × 0.32 mm × 0.5 μm, Agilent Technologies). The temperature programme was as follows: 40 °C (5 min), 20 °C∙min^−1^ to 250 °C, 250 °C (6 min).

For further analysis of data, it was assumed that the total analytical uncertainty is approximately ±0.5 ‰, so the observed fractionation must be at the minimum ±2 ‰ to confirm biodegradation (to ensure reliable interpretation, as presented in, e.g. Hunkeler et al. 2008). The isotope fractionation during volatilization, dissolution, diffusion and sorption is typically small and does not influence significantly the isotopic composition of the contaminants (Hunkeler et al. 2008). Hence, it was assumed that the isotope fractionation is caused solely by biodegradation.

## Results and discussion

### Batch tests

**Fig. 4:**
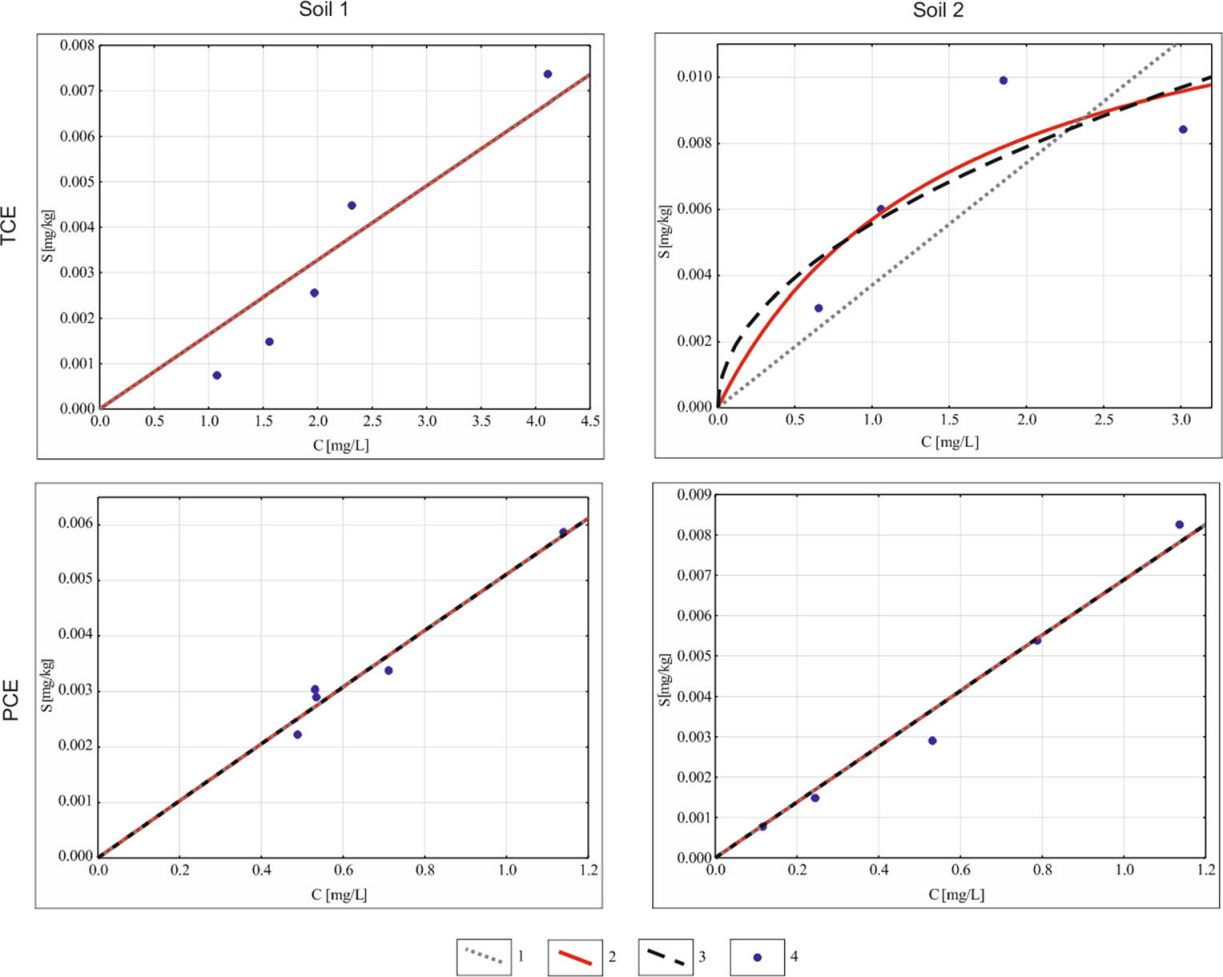
Sorption isotherms of TCE and PCE for investigated soils *1*—Henry’s, *2*—Langmuir’s, *3*—Freunlich’s, *4*—observed (measured) values

The time to reach equilibrium for soil 1 was of 30 and 40 days for TCE and PCE, respectively, i.e. 10 days shorter than for soil 2. The concentrations of TCE and PCE in solutions were fitted to three sorption models: Henry’s, Freundlich’s and Langmuir’s (Fig. 4). The determination coefficients (*r* ^2^) for considered sorption models are listed in Table 5. In the case of TCE, a sufficient fitting was obtained for the linear and Langmuir’s models: *r* ^*2*^ = 0.88 (soil 1) and for the Langmuir model: *r* ^*2*^ = 0.74 (soil 2). In the case of PCE, the best correlations were for the linear model: *r* ^*2*^ = 0.96 (soil 1) and *r* ^*2*^ = 0.98 (soil 2). Based on the fitted sorption models, retardation factors were calculated for both contaminants and aquifer materials (soils 1 and 2) (Table 6).

**Table 5:** Fitted isotherms and correlation coefficients for investigated soils

	Henry’s		Freundlich			Langmuir		
	*r* ^2^	*K*_*d*_	*r* ^2^	*K*_*f*_	*η*	*r* ^2^	*S*_max_	*K*_l_
TCE								
Soil 1	0.88	0.0016	–	–	–	0.88	1.8244	0.0009
Soil 2	0.21	0.0037	0.66	0.0056	0.5024	0.74	0.0145	0.6493
PCE								
Soil 1	0.96	0.0051	0.96	0.0051	0.9967	0.96	0.4763	0.0108
Soil 2	0.98	0.0069	–	–	–	0.98	1.5467	0.0045

*r* ^2^ determination coefficient [−], *K*_*d*_, *K*_*f*_, *K*_l_ partition coefficients, *η* degree of isotherm nonlinearity characterizing adsorption energy, *S*_max_ sorption capacity [mg/kg]

**Table 6:** Retardation factors R for TCE and PCE estimated from laboratory studies

Sample/parameter	R [−]	
	TCE	PCE
Batch tests		
Soil 1	1.01	1.31
Soil 2	1.34	1.41
Column experiments		
Soil 1	1.05	1.25
Soil 2	1.28	1.41
Soil 2 (TCE&PCE)	1.44	2.08

### Column experiments

**Fig. 5:**
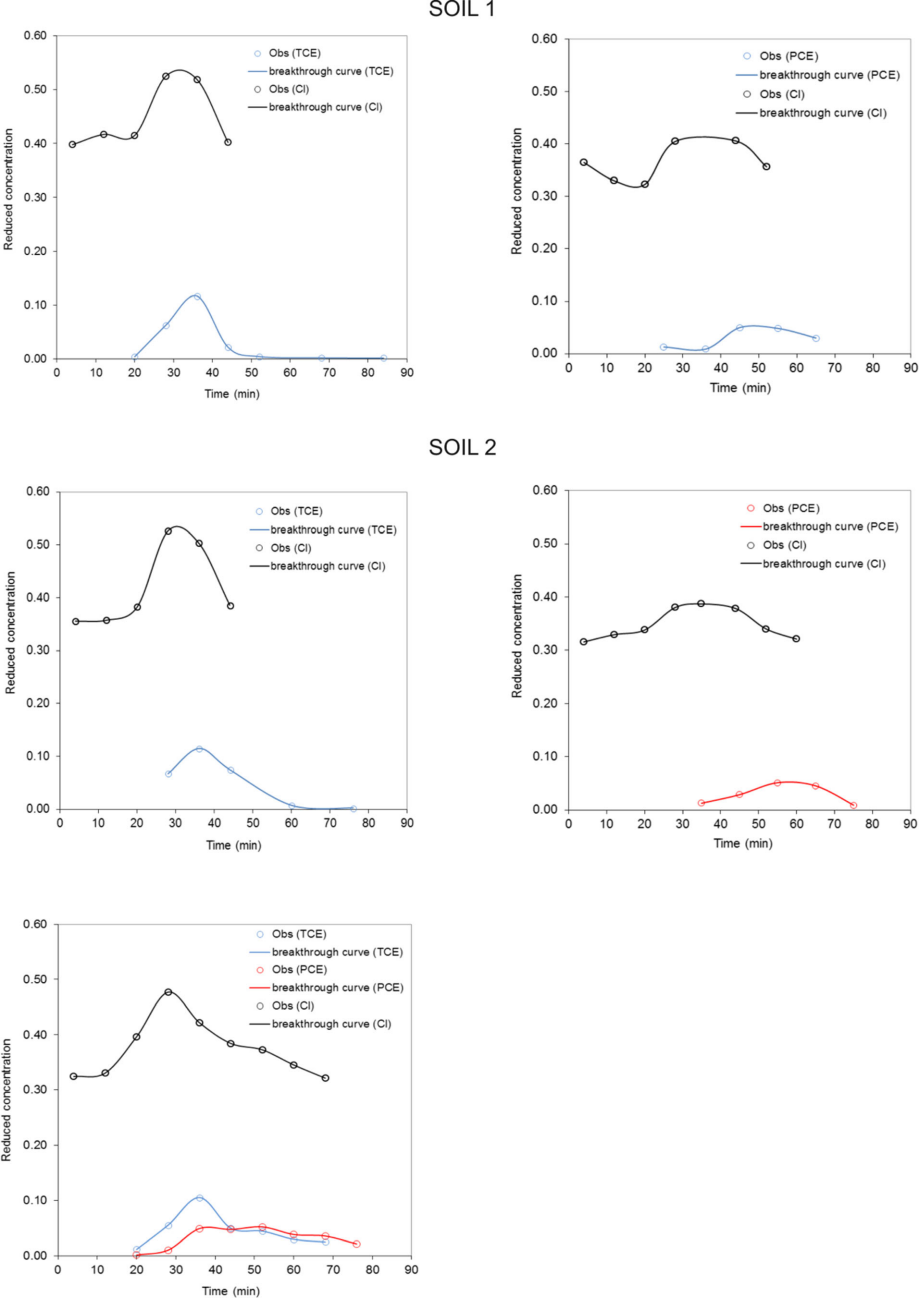
Observation (circles) and nonlinear least squares fits (lines) for soil 1 and 2

In the column experiment, 11 BTCs were registered (Fig. 5). Based on them, retardation coefficients (*R*) for both contaminants and aquifer materials were calculated (see Table 6). Since the samples were taken every ca. 8 min, the *R* values were calculated for the average time of sampling. The calculated *R* values for soil 2 are higher than for soil 1. They are also higher comparing to the batch test results. Furthermore, when both contaminants were present in the column, TCE influenced on PCE, so that the *R* values of both contaminants are significantly higher (Table 6). Column experiments are more accurate, thus the results represent better sorption processes in the aquifer.

Batch and column studies indicate that both materials (medium and fine sand) are characterized by higher retardation of PCE than of TCE in the studied sandy aquifer. Moreover, higher *R* values can be observed for fine sand (soil 2), which is probably due to higher content of grains with small diameter in comparison to medium sand (soil 1). Thus, sorption of TCE and PCE on both aquifer materials can be classified as ‘small sorption capacity’ according to the classification presented by Witczak et al. (2013) (Tables 7). Only when both contaminants are present together, the sorption capacity is between ‘small’ and ‘medium’. The results obtained are within the range of those reported in literature (see Table 2).

**Table 7:** Sorption capacity classification (Witczak et al., 2013, modified)

Sorption capacity	Retardation coefficient [−]
Small	1–2
Medium	2–10
Large	10–100
Very large	100–1000
Unlimited	>1000

**Table 8:** Results of the compound specific isotope analysis (CSIA)

Compound	Well name	Concentration [μg/L]	Sample size	Mean	95 % Confidence interval for mean		Standard deviation	Standard error
TCE	S2tr	807	9	−24.031	−24.430	−23.633	0.5186	0.1729
	M5	97	5	−23.901	−24.277	−23.525	0.3029	0.1354
	H2	968	12	−23.709	−23.982	−23.437	0.4290	0.1238
	S6b	42	13	−23.922	−24.314	−23.529	0.6495	0.1801
	S4c	107	9	−24.225	−24.926	−23.524	0.9120	0.3040
PCE	S2tr	387	8	−27.742	−27.991	−27.493	0.2981	0.1054
	M5	38	6	−27.149	−27.912	−26.386	0.7267	0.2967
	H2	51	5	−26.823	−28.486	−25.161	1.3389	0.5988

### Biodegradation studies

**Fig. 6:**
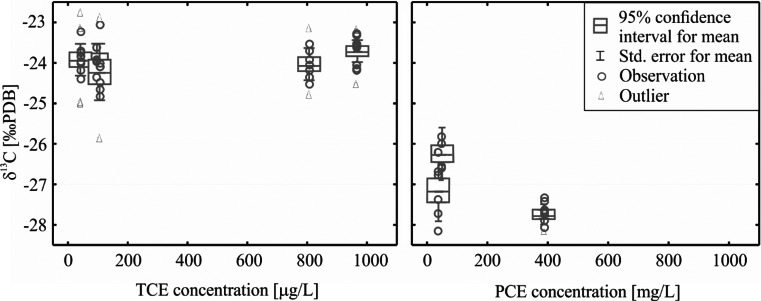
Box plots of δ^13^C

The maximum concentrations in analysed samples reached 807 and 387 [μg/L] for TCE and PCE, respectively. In two monitoring points (H-1, S-9) no TCE and PCE and in two others (S4c, S6b) no PCE were detected; therefore, no isotope analysis was performed in these points. For the remaining points. the measured average δ^13^C values were for TCE within the range of 23.6 ÷ −24.3 ‰ and for PCE of −26.3 ÷ 27.7 ‰ (Table 8, Fig. 6). In the case of TCE, the difference between the maximum and minimum δ^13^C may not be considered as significant, because it is less than ±1 ‰. For PCE, the difference is about ±1.4 ‰, still too low to clearly indicate biodegradation to occur. Nevertheless, PCE concentrations were mostly relatively low; therefore, the mean δ^13^C was calculated with higher standard deviation, so the reliability of δ^13^C is not sufficient enough (allowed standard deviation is ≤0,5 ‰). Neither the correlation of δ^13^C and the concentration of chlorinated solvents nor the distance from source was observed. Therefore, it was concluded that there is inadequate evidence for biodegradation of TCE and PCE via reductive dehalogenation to occur at the studied site. Hence, the main processes leading to the observed decrease of TCE and PCE concentrations in groundwater are dilution and sorption.

## Conclusions

Two experiments (batch and column) were used to estimate sorption of TCE and PCE in the sandy aquifer. Results obtained indicate that sorption (and consequently retardation) of both contaminants are low. Batch tests resulted in lower *K*_d_ and *R* values compared to the column experiment. Generally, TCE is characterized by lower retardation than PCE in the studied aquifer. Furthermore, in presence of both contaminants simultaneously in the column TCE influenced sorption of PCE, resulting in almost two times higher *R* values for both contaminants.

Estimated sorption parameters reflect the results described in literature for the similar types of experiments and soils. They indicate that transport of TCE and PCE in groundwater is controlled by properties of the aquifer materials, such as particle size and TOC.

There is an inadequate evidence for biodegradation of TCE and PCE via reductive dehalogenation to occur in the studied aquifer; thus, this parameter may be neglected in the modelling studies.

The results of the study allow for better understanding of TCE and PCE migration in the studied aquifer. They provide input parameters for numerical contaminant transport modelling to estimate risk for the receptors (municipal waterworks) and support the decision-making process for selecting an effective remediation strategy.

Results obtained in the presented study are used in fate and transport model that is recently being built using Visual MODFLOW software. MTD3MS code was selected in our study to model transport processes of chlorinated solvents in groundwater. For this code, sorption and reaction parameters are assigned on a cell-by-cell basis. As input parameters to characterize TCE and PCE transport in the aquifer, the following parameters were specified:

(i) The sorption isotherm type—Henry’s
(ii) the partition coefficient (*K*_d_) (calculated from *R* factor based on an isotherm type and corresponding equation), varying from 3.7°10^−11^ to 8.5°10^−11^ L/μg and from 1.21°10^−11^ to 2.10°10^−11^ L/μg for TCE and PCE, respectively (depending on the layer)
(iii) the first-order reaction (biodegradation) rate for the dissolved (or mobile) phase—omitted from the fate and transport model
